# Effect of Chemical Structure and Apparent Density of Rigid Polyurethane Foams on the Properties of Their Chemical Recycling Products

**DOI:** 10.3390/ma17246190

**Published:** 2024-12-18

**Authors:** Marcin Zemła, Maria Kurańska, Laima Vevere, Mikelis Kirpluks, Elżbieta Malewska, Maria Sofia Apostolou, Aleksander Prociak

**Affiliations:** 1 Faculty of Chemical Engineering and Technology, Cracow University of Technology, Warszawska 24, 31-155 Kraków, Poland; marcin.zemla@pk.edu.pl (M.Z.);; 2 Polymer Laboratory, Latvian State Institute of Wood Chemistry, Str. Dzerbenes 27, 1006 Riga, Latvia; 3 National Technical University of Athens, Heroon Polytechniou 9, 15780 Zografou, Greece

**Keywords:** chemical recycling, rebiopolyol, biopolyol, polyurethane foams

## Abstract

The aim of this work was to synthesize polyurethane foams based on petrochemical polyols and biopolyols with specific apparent densities (40, 60, 80, 100, and 120 kg/m^3^), test their properties, glycolyze them, and finally analyze each glycolyzed product. The petroleum-based foams, used as reference foams, and the bio-based foams underwent a series of standard tests to define their properties (the content of closed cells 20–95%, compressive strength 73–1323 kPa, thermal conductivity 24–42 mW/m∙K, brittleness 4.6–82.9%, changes in linear dimensions < 1%, and water absorption < 1%). Taking into account the need for recycling, the foams were shredded and then glycolyzed by diethylene glycol, with the addition of a catalyst in the form of potassium hydroxide. The chemolysis products were analyzed through determination, i.e., the amine and the hydroxyl values, viscosity, and molecular weight. The obtained rebiopolyols had hydroxyl numbers ranging from 476 to 511 mg KOH/g. The type of biopolyol used in the PUR foam systems had a significant impact on the amine number and the viscosity of the obtained rebiopolyol.

## 1. Introduction

Currently, polyurethanes (PURs) are one of the most important polymer materials. They are mainly used in construction, transport, and furniture [1]. The value of the PUR market in 2021 was estimated at USD 77.9 billion. According to forecasts, it will increase to USD 105.3 billion in 2026 [2]. Polyurethane foams are the most commonly produced PUR materials. The value of the global PUR foam production in 2020 amounted to USD 37.8 billion and is expected to increase to USD 54.3 billion in 2025. The production volume of PUR materials in 2022 reached almost 25.8 million tons and is forecasted to increase to 31.3 million tons in 2030, of which about 26% is the production of rigid polyurethane foam [3].

The constantly growing production of PUR foams requires consideration of how to manage PUR waste [4]. PUR foam waste stored in landfills takes up a lot of space because it has a very low apparent density. In the face of PUR storage space limitations, increasing storage costs, and restrictive legal requirements regarding environmental protection, it has become necessary to develop efficient and safe methods for recycling polyurethane waste and its subsequent management. Recycled PUR foams can be used in a variety of manufacturing processes, providing an alternative to currently used raw materials. Although the recycling of PURs is a technological and economic challenge, various methods can be used to enable this process. The two most important ways to recycle PUR foam waste are mechanical recycling and chemical recycling. The mechanical recycling of PUR foam waste is simple, cost-effective, convenient, and environmentally friendly [5]. One of the most effective ways to recycle PUR foams is to grind such materials and integrate them into a new product as a filler. The industry often employs such waste as a filler to produce new PUR materials, novel plaster materials, asphalts, cement, coatings, and adhesives [6,7]. The second way of managing PUR foam waste is chemical recycling. It involves partial degradation of the polymer structure, most often under the influence of other low-molecular compounds. A big problem in the chemical and mechanical recycling process of PUR foams is the fact that they are produced from different raw materials and characterized by different apparent densities, compressive strength, and flexibility [8]. For this reason, the products obtained after chemical recycling of such materials may differ in properties depending on the type of the material used. Just like in the case of mechanical recycling, products obtained through the chemical recycling of PUR foams can be used as raw materials in the production of new PUR materials [7].

The most commonly used chemical recycling methods are hydrolysis, acidolysis, and glycolysis [9]. They lead to the breaking of characteristic bonds in PURs, such as urethane and urea bonds. Degradation of the chemical structure of a polymer allows depolymerization of PUR waste into oligomers terminated with reactive groups. Then, the material can be used further as a polyol component for the synthesis of new PUR materials. The use of the above-mentioned chemical recycling methods allows for the obtaining of repolyols containing hydroxyl and amine groups, which are able to react with isocyanate components in new PUR systems [10]. The glycolysis of PUR waste has to be carried out in a reactor with a mechanical stirrer at temperatures ranging from 150 °C to 220 °C, unlike, e.g., hydrolysis, which requires temperatures between 200 °C and 340 °C and increased pressure. For this reason, the glycolysis of PUR waste is less energy-consuming and easier than hydrolysis. The heating of the reaction mixture can be carried out conventionally or by using microwave irradiation. The use of microwave radiation significantly shortens the glycolysis process compared to conventional heating [11,12].

In the present study, rigid PUR foams with different apparent densities have been obtained using a petrochemical polyol and a biopolyol synthesized from rapeseed oil by transesterification with triethanolamine. Then, the foams were subjected to chemical recycling using diethylene glycol. The influence of apparent density and polyol type on the properties of rigid PUR foams and their derivative glycolysates was analyzed.

## 2. Materials and Methods

### 2.1. Polyurethane Synthesis and Testing

#### 2.1.1. Raw Materials

**Figure 1:**
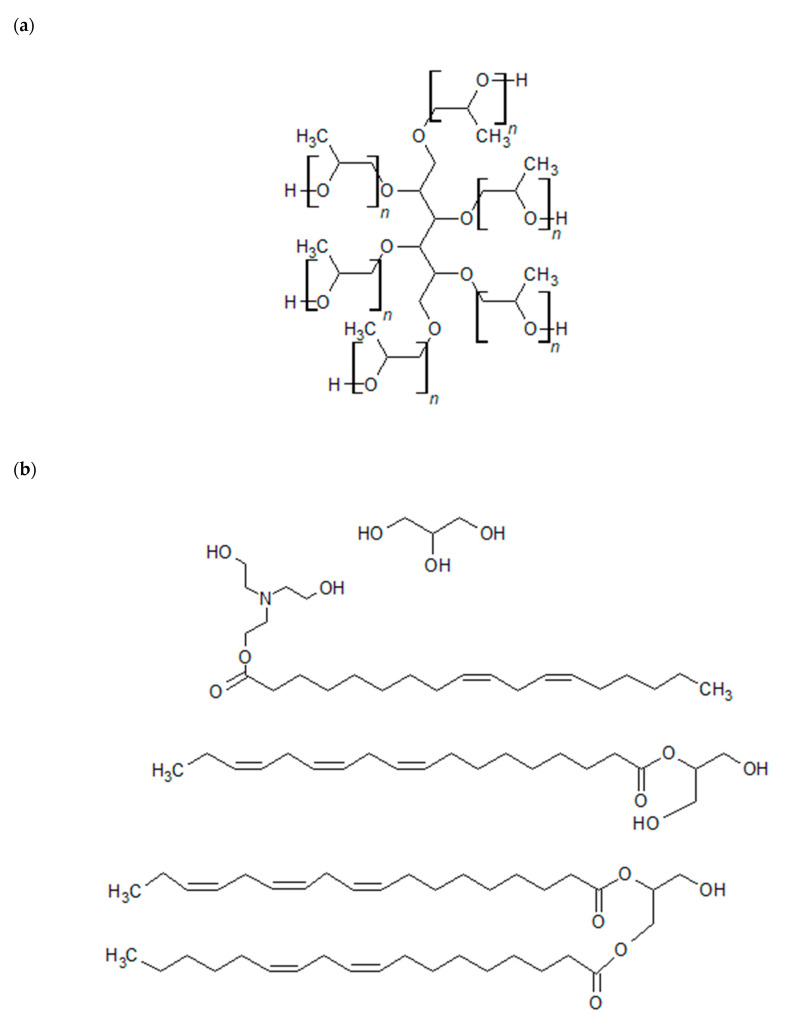
Chemical formulas of used polyols: (**a**) RF-551 and (**b**) TRE_TEA.

The raw materials used in the research were petrochemical polyol Rokopol^®^ RF-551 (hydroxyl number: 405 mgKOH/g, viscosity: 3030 mPa·s (25 °C), water content: 0.1%) provided by PCC Rokita S.A, Brzeg Dolny, Poland; biopolyol TRE_TEA (hydroxyl number: 416 mgKOH/g, viscosity: 60 mPa·s (25 °C), water content: 0.246%) synthesized in the Department of Chemistry and Technology of Polymers at the Cracow University of Technology (Kraków, Poland) by the transesterification of rapeseed oil with triethanolamine (Figure 1); EKOPUR B (PMDI) (isocyanate group content: 31 wt.%, viscosity: 200 mPa·s (25 °C)) supplied by Minova Ekochem S.A Poland (Siemianowice Śląskie, Poland); catalyst NIAX™ A-1 supplied by Momentive Performance Materials; and surfactant Niax SR-321 supplied by Momentive Performance Materials (Niskayuna, NY, USA). 

#### 2.1.2. Polyurethane Synthesis

**Figure 2:**
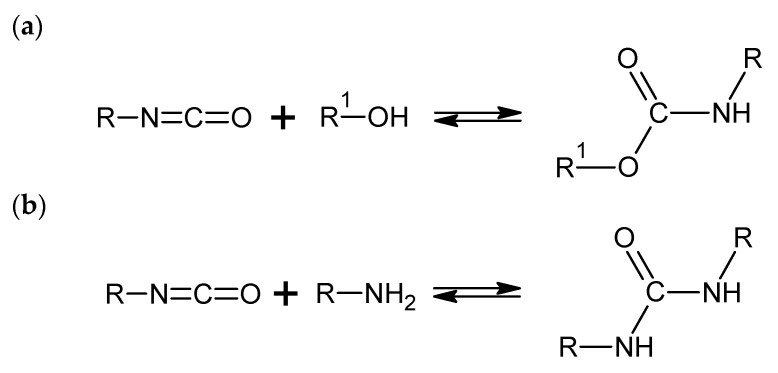
Basic reactions during the foaming process: (**a**) formation of a urethane group and (**b**) formation of a urea group.

Rigid PUR foams were manufactured following a one-step method by mixing a polyol premix (polyol, catalyst, surfactant, and blowing agent) with isocyanate and then pouring the reaction mixture into an open mold. The preparation of the first group of foams involved petrochemical polyol RF-551, catalyst Niax A-1, surfactant Niax SR-321, water, and isocyanate PMDI. The preparation of the second group of foams required all the components listed above except for the petrochemical polyol, which was replaced by a biopolyol synthesized from rapeseed oil by transesterification with triethylamine. The synthesis steps were the same for each system. A polyol premix containing the right amount of polyol, catalyst, surfactant, and water was prepared and homogenized by mixing. After that, PMDI was added to the mixture and mixed for 5 s by a mechanical stirrer. The reactive system was then poured into an open mold, which allowed it to rise freely in a vertical direction. The system was left for 24 h to let the reaction come to an end. The isocyanate index (INCO) of all the PUR foams obtained was 110. The symbols and formulations of the polyurethane systems are shown in Table 1. Basic reactions during the foaming process were shown in Figure 2.

#### 2.1.3. Foam Testing Methods

The characteristic processing times, such as gel time, tack-free time, and rise time, were recorded by a stopwatch during the foaming process. Apparent densities were measured according to ISO 845 [13]. The content of closed cells was found according to ISO 4590 [14] by cutting out samples with dimensions of around 25 × 25 × 100 mm^3^ and measuring their volumes. The sample was placed in a chamber whose volume was increased by a constant value. The pressure drop was measured on the scale of an open-tube manometer. Based on calibration with metal standards, the impenetrable volume of the foam was calculated. Thermal conductivity was determined at an average temperature of 10 °C (hot plate 20 °C, cold plate 0 °C) using a Laser Comp Heat Flow Instrument Fox 200 according to ISO 8301 [15]. The dimensional stability of the foams was determined using samples with dimensions 100 × 100 × 25 mm^3^. Initial measurements of the length, width (perpendicular to the foam rise direction), and thickness (parallel to the foam rise direction) were taken. Subsequently, the samples underwent a 24 h period of freezing at −25 °C, after which the measurements were taken again. The samples were subjected to a 24 h exposure in an oven set at 70 °C, and the dimensions were measured once more according to ISO 2796-1986 [16]. Water absorption for each foam was determined as an average of four samples after immersion in distilled water for 24 h, according to ISO 2896 [17]. The compressive strength was tested following ISO 844 [18], involving measurements at 10% deformation and measurements of the Young modulus. The specimens tested were cut into cylindrical shapes with diameters and heights of 40 mm, both parallel and perpendicular to the foam rise direction. Brittleness testing involved cutting out cubes (25 × 25 × 25 mm^3^) from each foam system and placing them in a rotating box with wooden cubes, where the cubes experienced collisions. The masses of 12 cubes were measured before and after rotations to assess the weight loss according to ASTM C-421 [19].

#### 2.1.4. Chemical Recycling of PUR Foams

PUR from each system was collected and then shredded. The chemolysis agent used for the recycling of the PUR foams was diethylene glycol (DEG), and the catalyst used was potassium hydroxide (KOH). The reactants consisted of DEG and PUR in a 1:1.5 ratio. The mass of KOH was chosen to be 1% of the DEG mass. The mixture of DEG and KOH was transferred to a round-bottom flask, which underwent mechanical stirring and heating via a mantle set to 180 °C. The powdered PUR was gradually added to the flask over 2–3 h. After adding the entire mass of PUR, the reaction was carried out for 2 h at 180 °C. The obtained repolyols (glycolysates of petrochemical foams) and rebiopolyols (glycolysates of biofoams) were analyzed without further purification.

#### 2.1.5. Analysis of Repolyols and Rebiopolyols

Amine values were analyzed according to BN-69/6110–29 [20]. A sample of glycolysate (approx. 0.3 g) was dissolved in a mixture of 40 cm^3^ of acetone and 10 cm^3^ of demineralized water. After dissolving and adding bromocresol green as an indicator, the sample was titrated with hydrochloric acid (0.1 mol/dm^3^) until the color changed to yellow.

Hydroxyl values were determined according to ISO-4629-2 [21]. The analysis involved a reaction of excess acetic anhydride with hydroxyl groups in glycolysates and then titration of acetic acid resulting from the decomposition of acetic anhydride using a sodium hydroxide solution (1 mol/dm^3^) in the presence of thymolphthalein.

The viscosity of repolyols and rebiopolyols was determined using a rotational rheometer RM200 CP4000 PLUS (Lamy Rheology, Champagne-au-Mont-d'Or, France). Analyses were performed in a plate-plate configuration at a temperature of 25 °C using a 20 mm diameter plate, a 1 mm gap, and a rotational speed of 100 rpm.

The average molecular weight number (Mn), weight average molecular weight (Mw), dispersity (D), and functionality (f) of glycolysates were determined using gel permeation chromatography using a Knauer chromatograph (Berlin, Germany). During the analysis, the eluent was stabilized tetrahydrofuran, the flow of which was set at 1 mL/min.

Repolyols and rebiopolyols were analyzed by FTIR spectroscopy using a Nicolet iS5 spectrometer (Thermo Fisher Scientific, Waltham, MA, USA). Spectra were recorded using a diamond ATR fixture in the range of 4000–500 cm^−1^ with 20 scans per spectrum and a spectral resolution of 4 cm^−1^. 

## 3. Results and Discussion

### 3.1. Polyurethane Foams and Biofoams

The reactivity of PUR systems was analyzed. For this purpose, characteristic processing parameters during the foaming PUR systems were measured. Table 2 displays the results concerning gel time, tack-free time, and rise time. In petroleum-based foams, gel time, rise time, and tack-free time tend to increase with apparent density. This is due to the fact that obtaining higher-density foam requires the use of less water as a blowing agent, which means that less heat is released in the highly exothermic reaction of water with the isocyanate component. This means that less water in the PUR system reduces reaction speed. Gel time ranges from 45 to 95 s, rise time ranges from 64 to 109 s, and tack-free time ranges from 86 to 140 s. In contrast, the changes in characteristic processing times with water content in the systems containing also biopolyols were only slight. In this case, the decrease in water amount can be minimized by a highly reactive biopolyol containing amine groups that catalyze the reaction of obtaining a polyurethane [22,23,24]. So, the heat of the exothermic reaction is released in a shorter time. The bio-based foams exhibit gel times ranging from 18 to 21 s, rise times from 33 to 41 s, and tack-free times from 35 to 51 s.

The content of closed cells is an important parameter characterizing rigid PUR foams. To obtain a material with good thermal insulation properties, a high content of closed cells is expected, preferably above 90%. This allows gas with a low thermal conductivity coefficient to be enclosed in the foam cells, thus limiting convection in heat transport.

The content of closed cells in all the foams manufactured from the petrochemical polyol was high, 91.5% (Table 3). A decrease in apparent density did not significantly affect the closed cell content of these PUR foams. Concerning the bio-based foams, the content of closed cells was much lower than the one of the petroleum-based foams, ranging from 15.9 to 53.9%. The errors in the measurements are noticeably higher in this case, which may result from the high reactivity of the biopolyol. In its structure, the biopolyol contains amine groups, which catalyze the polyurethane formation reaction [25,26]. The high error values for the bio-based foams suggest data inconsistency, indicating variations in closed cell content within different areas of the foam. The closed cell content of biofoams decreases with decreasing apparent density, which may be related to thinner cell walls due to fewer polyurethane materials in the unit of volume. It causes thinner walls of cells, which have the tendency to crack more easily during the foaming process. In the case of the BioPUR foams, the low values of closed-cell content significantly influenced the thermal conductivity. It was caused by a replacement of carbon dioxide (created in the reaction of isocyanate with water) with air characterized by higher gas thermal conductivity.

The results on thermal conductivity are displayed in Table 3. An increase in the apparent density causes an increase in the heat conduction coefficient because an increase in the apparent density leads to a significant increase in the heat conduction through the polymer matrix [27]. Notably, the thermal conductivity of the bio-based foams exhibits significantly higher values, ranging from 37.01 to 42.30 mW/m·K, while the petroleum-based polyols demonstrate lower values from 24.09 to 30.23 mW/m·K. Higher values of the thermal conductivity coefficient of the foams modified with the biopolyol may come from a low content of closed cells in the material.

Table 4 presents the results of compressive strength measurements at 10% deformation and the Young modulus of the PUR foams for directions both parallel and perpendicular to the foam rise direction. When comparing materials with equivalent apparent densities, it becomes evident that for both the petroleum-based and bio-based foams, the compressive strength values in a parallel direction are consistently higher than those recorded in a perpendicular direction, which results from an anisotropic structure of PUR foam cells. Moreover, regardless of the direction, the compressive strength increases with a growing apparent density. When comparing pairs of petroleum-based and bio-based foams at similar apparent densities, the former exhibit consistently higher values. For the petroleum-based foams, the compressive strength in parallel rise ranges from 241 to 1323 kPa, while in perpendicular rise, its range is 176 to 1122 kPa. On the other hand, for the bio-based foams, the parallel rise compressive strength ranges from 100 to 468 kPa, and in a perpendicular direction, it ranges from 73 to 326 kPa.

As for the Young modulus, the same comparisons were made between directions parallel and perpendicular to the foam rise direction, looking at materials with equivalent apparent densities within the two foam groups. The Young modulus values range from 7.10 to 41.35 MPa in a parallel rise direction and from 4.80 to 32.22 MPa in a perpendicular rise direction for the petroleum-based foams. For the bio-based foams, the Young modulus ranges from 4.36 to 18.89 MPa in a parallel rise direction and from 1.44 to 7.07 MPa in a perpendicular rise direction. Significantly worse mechanical properties of the biofoams compared to the respective foams with petrochemical polyols are the result of the plasticizing effect of biopolyols from vegetable oils. This is due to the presence of long carbon chains in biopolyol. The plasticizing effect is due to the presence of long carbon chains (especially dangling fragments derived from fatty acids) in the biopolyol [28].

The influence of apparent density on the brittleness of the rigid PUR foams obtained from the petrochemical polyol and those derived from the biopolyol is completely different. The apparent density of the PUR foams obtained using the petrochemical polyol does not significantly affect their brittleness, which ranges from 4.6 to 5.8% (Table 5). The brittleness of the PUR foams obtained from the rapeseed oil polyol is much greater than that of the foams obtained from the petrochemical polyol, ranging from 33.6% to 82.9%. The highest brittleness of 82.9% occurs at 60 kg/m^3^ and decreases for higher apparent densities. The high brittleness of the biofoams may be due to the low mechanical strength and content of closed cells and a lower average molecular weight of the biopolyol compared to the petrochemical polyol [29]. The water absorption of the petrochemical-based PUR foams increases with an increasing apparent density from 0.45% to 0.73%. On the other hand, the water absorption of the biobased PUR foams does not depend significantly on the apparent density of the foam materials. Such water absorption is suitable for PUR foams used as thermal insulation.

The dimensional stability of the rigid PUR foams was analyzed by changing their linear dimensions perpendicular to the direction of foam rise (A1, A2) and parallel to the direction of foam rise (B) after conditioning them at a temperature of −25 °C and 70 °C for 24 h (Table 6 and Table 7). In the petroleum-based foams, it is evident that the dimensions of the samples expanded. Notably, the expansion is more pronounced at 70 °C, with significantly higher values observed at equivalent apparent densities as compared to the situation seen at −25°C. In contrast, in some cases, the bio-based foams exhibit dimension shrinkage, particularly in thickness, at − 25 °C. On the other hand, at 70 °C, they exhibit expansion reaching 0.66%. Most importantly, the dimensions of the PUR foams after conditioning at temperatures of −25 °C and 70 °C for 24 h did not change by more than 0.7%, which means that they are dimensionally stable [30].

### 3.2. Analysis of Glycolysis Products of Polyurethane Foams

In the next stage, selected properties of the petroleum-based foams (repolyols) and the glycolysates of the bio-based foams (rebiopolyols) were studied. No significant effect of the PUR foam's apparent density on the hydroxyl and amine values was observed for the repolyols and rebiopolyols (Table 8). However, when comparing the glycolysis products of the PUR foams obtained from different polyols, it can be seen that the rebiopolyols have slightly lower hydroxyl values (476–489 mgKOH/g) than the repolyols (485–511 mgKOH/g). The methods of obtaining glycolysates of rigid and flexible PUR foams using diethylene glycol with similar hydroxyl number values have been described in other articles [4,11,31,32]. The amine numbers are much higher in the case of the rebiopolyols. This is due to the use of biopolyols containing amine groups in their structures. A higher amine number will increase the reactivity of a rebiopolyol in a polyurethane system because amine groups catalyze the reactions of polyurethane formation.

The repolyols and rebiopolyols were analyzed using gel permeation chromatography (GPC) (Table 9). In most cases, the average molecular weight number (Mn) and weight average molecular weight (Mw) increase with an increasing apparent density of a recycled PUR foam in the range from 642 g/mol to 710 g/mol (Mn) and from 1173 g/mol to 1333 g/mol (Mw). However, such a relationship was not observed for the rebiopolyols. The values of Mn and Mw stayed at a similar level when the apparent densities increased, in the range of 575–588 g/mol and 1028–1065 g/mol, respectively. The differences in the dispersity (D) of the obtained repolyols and rebiopolyols were not significant (1.8–1.9). In the case of repolyol Rec/PUR_40, it was found that the functionality (f) was lower than those seen for the other repolyols, which is due to the lowest Mn value of this glycolysate among the repolyols. However, for the rebiopolyols, the functionality slightly decreased with an increasing apparent density of the recycled PUR foams. In general, the functionality (f) of the repolyols was higher than that of the rebiopolyols, which may indicate greater chain branching in the repolyols.

**Figure 3:**
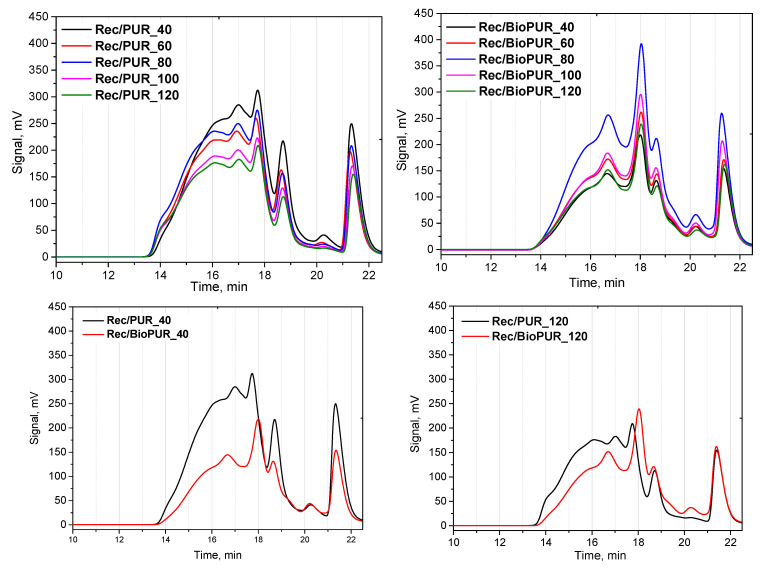
GPC chromatograms of repolyols and rebiopolyols.

Figure 3 shows GPC chromatograms of the obtained repolyols and rebiopolyols. It can be seen that the repolyols contain more chains with higher molecular weight compared to the rebiopolyols. This conclusion is supported by significantly larger and broader peaks, with maxima occurring between the 15th and 17th minute of testing, corresponding to the presence of the longest chains. Also, the repolyols and rebiopolyols contain diethylene glycol (DEG), which was used as a glycolysis agent. This is evidenced by the peak in the 21st minute. The amount of unreacted DEG in the repolyols was found to be between 10.6 and 11.4%, while in the rebiopolyols, it was 12.0–14.3%. The use of such products in polyurethane systems does not require further purification.

**Figure 4:**
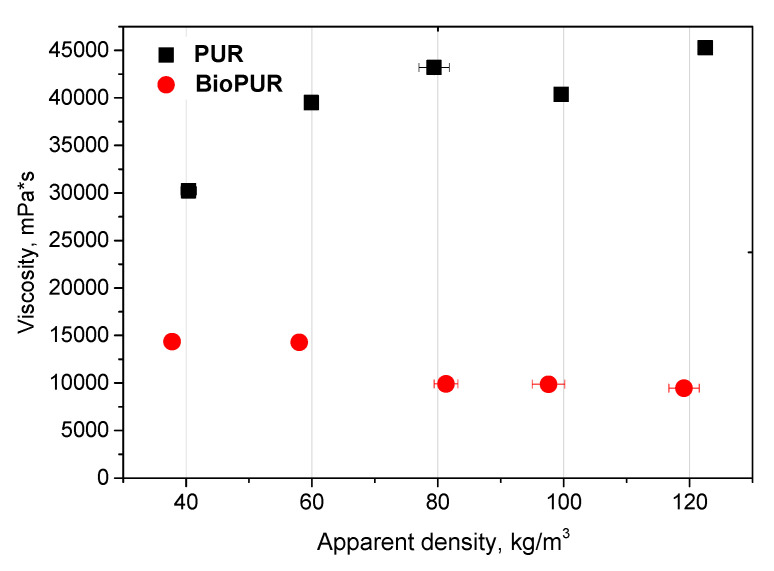
Correlation of viscosity of repolyols and rebiopolyols with apparent densities of recycled PUR foams.

The viscosity of the repolyols and rebiopolyols was analyzed by a rotational rheometer (Figure 4). The repolyols are generally more viscous than the rebiopolyols. This may be the result of shorter chains in the rebiopolyols than in the repolyols, as indicated by their significantly lower Mn and Mw values. Additionally, the viscosity of most repolyols increases with the apparent densities of the recycled PUR foams from 31,600 mPa∙s to 47,300 mPa∙s. The observed viscosity rise in the repolyols can be explained by increasing Mn, Mw as well as f, leading to the formation of larger branches and entanglement of repolyol chains. On the other hand, the increase in the apparent densities of the recycled biofoams resulted in a decrease in the viscosity of the rebiopolyols from 15,000 mPa∙s to 10,100 mPa∙s. In this case, the main cause may be the decrease in f and the decrease in the amount of branching and chain entanglement in the rebiopolyols.

**Figure 5:**
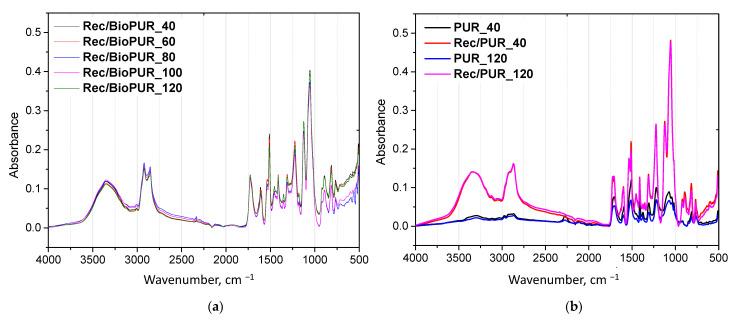
FTIR spectra of (**a**) rebiopolyols and (**b**) repolyols.

An FTIR analysis was used to confirm the presence of characteristic groups in the repolyols and rebiopolyols (Figure 5). FTIR spectra do not differ significantly across the recycled foams. All of the obtained glycolysates had a band with a wavenumber at about 3340 cm^−1^ corresponding to the characteristic stretching vibration of hydroxyl and N−H groups [33,34]. Bands with wavenumbers 2915 cm^−1^ and 2870 cm^−1^ represent the stretching vibration of CH_2_ groups in the carbon chain [32]. Bands at 1725 cm^−1^ and 1709 cm^−1^ correspond to the C=O from urethane and urea bonds [4,32]. The aromatic rings coming from the isocyanate component correspond to a band with a wavenumber of 1596 cm^−1^ [30]. Characteristic coupling band of symmetric deformation vibrations of the –N–H bonded groups with the stretching vibrations of the –C–N groups, deformation vibrations of the N−H groups correspond to the wavenumber 1537 cm^−1^ [4,35]. The band at 1513 cm^−1^ corresponds to the bending vibrations of N−H groups from urethane bonds [34]. Signals coming from bending vibrations of C−H in the polyol chain were at 1454 and 1374 cm^−1^ [32,36]. A band with a wavenumber 1222 cm^−1^ was due to the deformation vibration of –N–H with the tension vibration of –C–N in the urethane group [4,35]. The C–O–C vibrations in DEG and petrochemical polyol correspond to wavenumbers 1056 cm^−1^ and 1123 cm^−1^ [32].

## 4. Conclusions

This investigation delves into the properties of both petroleum-based and bio-based PUR foams and the products of their glycolysis. The findings provide valuable insights, particularly regarding correlations between selected properties and apparent densities, enabling a straightforward performance comparison between the bio-based and petroleum-based foams. The thermal conductivity increased with the rising apparent densities of the foams. The bio-based foams exhibited notably higher thermal conductivity values due to lower contents of closed cells in comparison to the petroleum-based foams. Furthermore, compressive strength at 10% deformation displayed a consistent upward trend with increasing apparent densities across all PUR foams. The petroleum-based foams had higher compressive strength than the bio-based foams in both parallel and perpendicular directions with respect to the foam rise direction, as also attested by the Young modulus results.

As far as the glycolyzed products are concerned, the rebiopolyols exhibited significantly higher amine values, indicating more reactive products than the repolyols, while their hydroxyl values fell down to the range that allows their use in new PUR systems. The repolyols had a higher viscosity than the rebiopolyols. The viscosity of the repolyols increased with an increase in the apparent densities of the recycled foams, which was a result of a rise in average molecular weights. In contrast, the viscosity of the rebiopolyols decreased as the apparent densities of recycled foams became higher.

## Figures and Tables

**Table 1:** Formulations of polyurethane systems.

PUR Symbol	RF551, php	TRE_TEA, php	A-1, php	SR321, php	Water, php	INCO
PUR_40	100	0	1.6	1.5	3.40	110
PUR_60					2.00	
PUR_80					1.45	
PUR_100					1.10	
PUR_120					0.87	
BioPUR_40	0	100	0.5		3.10	
BioPUR_60					1.65	
BioPUR_80					1.01	
BioPUR_100					0.82	
BioPUR_120					0.61	

php—parts per hundred polyol.

**Table 2:** Gel time, rise time, and tack-free time of PUR foaming processes.

Foam Symbol	Gel Time, s	Rise Time, s	Tack-Free Time, s
PUR_40	45 ± 2	64 ± 2	86 ± 1
PUR_60	59 ± 2	88 ± 2	102 ± 1
PUR_80	69 ± 2	109 ± 2	129 ± 2
PUR_100	85 ± 2	104 ± 1	133 ± 2
PUR_120	95 ± 2	109 ± 2	140 ± 1
BioPUR_40	21 ± 1	41 ± 2	51 ± 2
BioPUR_60	21 ± 1	38 ± 3	48 ± 2
BioPUR_80	18 ± 1	34 ± 2	36 ± 1
BioPUR_100	20 ± 1	36 ± 2	42 ± 2
BioPUR_120	21 ± 1	38 ± 2	38 ± 2

**Table 3:** Content of closed cells, thermal conductivity coefficient, and apparent density of PUR foams.

Foam Symbol	Content of Closed Cell, %	Thermal Conductivity Coefficient, mWm·K	Apparent Density, kg/m^3^
PUR_40	91.5 ± 1.4	24.09 ± 0.09	40.4 ± 1.2
PUR_60	92.0 ± 1.3	25.26 ± 0.21	59.9 ± 0.5
PUR_80	94.5 ± 2.2	26.37 ± 0.30	79.4 ± 2.4
PUR_100	93.1 ± 2.5	28.15 ± 0.07	99.6 ± 0.8
PUR_120	93.7 ± 1.9	30.23 ± 0.15	122.5 ± 0.9
BioPUR_40	20.4 ± 2.2	37.01 ± 0.12	37.8 ± 0.8
BioPUR_60	15.9 ± 12	38.79 ± 0.12	58.0 ± 0.9
BioPUR_80	19.5 ± 6.8	39.74 ± 0.08	81.3 ± 1.9
BioPUR_100	34.5 ± 2.5	40.45 ± 0.35	97.6 ± 2.6
BioPUR_120	53.9 ± 13	42.30 ± 0.16	119.1 ± 2.4

**Table 4:** Compressive strength at 10% of deformation, Young modulus of PUR foams.

Foam Symbol	Compressive Strength, Parallel, kPa	Young Modulus, Parallel, MPa	Compressive Strength Perpendicular, kPa	Young Modulus, Perpendicular, MPa
PUR_40	241 ± 10	7.10 ± 0.19	176 ± 13	4.80 ± 0.45
PUR_60	435 ± 18	13.56 ± 0.77	317 ± 11	8.63 ± 0.80
PUR_80	655 ± 20	21.11 ± 0.59	519 ± 22	14.61 ± 0.54
PUR_100	975 ± 15	30.47 ± 0.33	815 ± 25	23.59 ± 0.5
PUR_120	1323 ± 40	41.35 ± 0.91	1122 ± 42	32.22 ± 0.79
BioPUR_40	100 ± 15	4.36 ± 0.42	73 ± 4	1.44 ± 0.07
BioPUR_60	101 ± 16	4.45 ± 0.75	79 ± 9	1.50 ± 0.44
BioPUR_80	202 ± 18	9.98 ± 0.87	119 ± 17	2.22 ± 0.32
BioPUR_100	268 ± 19	12.70 ± 0.78	180 ± 5	3.65 ± 0.33
BioPUR_120	468 ± 34	18.89 ± 0.81	326 ± 13	7.07 ± 0.90

**Table 5:** Water absorption and brittleness of polyurethane foams.

Foam Symbol	Water Absorption, %	Brittleness, %
PUR_40	0.45 ± 0.05	4.6 ± 0.1
PUR_60	0.47 ± 0.04	5.8 ± 0.0
PUR_80	0.64 ± 0.04	5.3 ± 0.1
PUR_100	0.71 ± 0.03	5.5 ± 0.0
PUR_120	0.73 ± 0.05	4.6 ± 0.1
BioPUR_40	0.92 ± 0.04	69.8 ± 0.5
BioPUR_60	0.81 ± 0.05	82.9 ± 0.2
BioPUR_80	0.77 ± 0.04	78.6 ± 0.3
BioPUR_100	0.99 ± 0.03	48.5 ± 0.7
BioPUR_120	0.73 ± 0.03	33.7 ± 0.5

**Table 6:** Dimensional stability of PUR foams at −25 °C.

Foam Symbol	A1, %	A2, %	B,%
PUR_40	0.09 ± 0.10	0.07 ± 0.10	0.35 ± 0.27
PUR_60	0.12 ± 0.04	0.09 ± 0.04	0.21 ± 0.20
PUR_80	0.07 ± 0.04	0.05 ± 0.02	0.18 ± 0.14
PUR_100	0.05 ± 0.03	0.06 ± 0.03	0.15 ± 0.26
PUR_120	0.06 ± 0.04	0.05 ± 0.03	0.00 ± 0.12
BioPUR_40	−0.14 ± 0.17	0.00 ± 0.21	−0.29 ± 0.73
BioPUR_60	0.03 ± 0.19	0.10 ± 0.18	−0.44 ± 0.59
BioPUR_80	−0.03 ± 0.09	−0.05 ± 0.26	−0.23 ± 0.37
BioPUR_100	0.06 ± 0.06	0.12 ± 0.12	−0.16 ± 0.16
BioPUR_120	0.06 ± 0.04	0.01 ± 0.06	−0.09 ± 0.07

**Table 7:** Dimensional stability of PUR foams at 70 °C.

Foam Symbol	A1, %	A2, %	B,%
PUR_40	0.39 ± 0.07	0.46 ± 0.11	0.50 ± 0.24
PUR_60	0.21 ± 0.05	0.24 ± 0.06	0.46 ± 0.20
PUR_80	0.17 ± 0.05	0.19 ± 0.03	0.40 ± 0.19
PUR_100	0.22 ± 0.06	0.20 ± 0.08	0.35 ± 0.12
PUR_120	0.18 ± 0.06	0.19 ± 0.04	0.44 ± 0.12
BioPUR_40	0.53 ± 0.18	0.49 ± 0.18	0.62 ± 0.52
BioPUR_60	0.14 ± 0.24	0.08 ± 0.12	0.66 ± 0.34
BioPUR_80	0.02 ± 0.15	0.02 ± 0.27	0.40 ± 0.38
BioPUR_100	−0.05 ± 0.05	−0.10 ± 0.10	0.18 ± 0.18
BioPUR_120	−0.18 ± 0.13	−0.16 ± 0.08	0.24±0.12

**Table 8:** Hydroxyl and amine values of repolyols and rebiopolyols.

Sample Symbol	Hydroxyl Value, mgKOH/g	Amine Value, mgKOH/g
Rec/PUR_40	511 ± 4	9.49 ± 0.43
Rec/PUR_60	491 ± 9	7.64 ± 0.06
Rec/PUR_80	493 ± 3	7.31 ± 0.30
Rec/PUR_100	493 ± 4	6.35 ± 0.30
Rec/PUR_120	485 ± 5	7.58 ± 0.03
Rec/BioPUR_40	485 ± 1	32.89 ± 0.08
Rec/BioPUR_60	476 ± 1	34.88 ± 0.06
Rec/BioPUR_80	480 ± 3	35.23 ± 0.57
Rec/BioPUR_100	489 ± 4	38.61 ± 0.64
Rec/BioPUR_120	482 ± 4	35.50 ± 0.42

**Table 9:** Gel permeation chromatography results.

Sample Symbol	Mn, g/mol	Mw, g/mol	D	f
Rec/PUR_40	642	1173	1.8	5.8
Rec/PUR_60	697	1291	1.9	6.1
Rec/PUR_80	706	1327	1.9	6.2
Rec/PUR_100	695	1306	1.9	6.1
Rec/PUR_120	710	1333	1.9	6.1
Rec/BioPUR_40	578	1045	1.8	5.0
Rec/BioPUR_60	588	1065	1.8	5.0
Rec/BioPUR_80	578	1048	1.8	4.9
Rec/BioPUR_100	576	1028	1.8	4.9
Rec/BioPUR_120	575	1069	1.9	4.9

## Data Availability

The original contributions presented in this study are included in the article. Further inquiries can be directed to the corresponding author.
